# The Importance of a Correct Medico-Dental Education

**Published:** 1849-01

**Authors:** J. S. King

**Affiliations:** Lancaster, Ohio


### THE IMPORTANCE OF A CORRECT MEDICO-DENTAL EDUCATION.

BY J. S. KING, D. D. S.—*Lancaster, Ohio.*

THE subject of a proper dental standard of attainment is one which, judging from daily observation, has not received that attention from the profession which it deserves, and which the interest of the science as a distinct profession, and the good of the public demand.

Why, let me ask, should Dr. A. require a pupilage of two or three years, Dr. B. one or two, and Mr. C. only five or six months; and again, Mr. D., who claims superior skill and most profound erudition, hoots at the idea of a longer pupilage than five or six weeks. I find the tuition fee does not thus vary, for all ask near the same.

There must be some cause for this most singular diversity of principle and practice. Let us see if the cause can be properly ascertained.

The profession—that is, the different members who constitute the profession *en masse*—cannot all alike appreciate the importance of dental operations, neither can they alike regard the difficulties to be overcome in the varied manipulations necessary to their proper performance; for if this was the case, and they felt a proper regard for the profession itself, their views would be similar; and laying aside the different capacities which may exist in different students, all would demand about the same term of study. Take it for granted, then, that all are alike honest, and it is apparent that some must have capacities for acquiring knowledge five times greater than others, and measure the standard of their students by themselves; or they must view study and skill of very little avail in this department of surgery. They must also have very different conceptions of what constitutes good operations. I am satisfied that superior capacities cannot make up for the discrepancy which exists as it regards the necessary term of study; and observation has led me to believe that the cause lies in these two things: First, what constitutes good operations; and, second, what amount of knowledge is requisite to guide us aright in their performance.

As it regards the first proposition, I would remark, that there is a greater difference than is generally supposed, and that this is not so much manifest in *theory* as in *practice*. For instance, I find that Dr. A. and Mr. D. talk about the same—both deplore the great amount of quackery in the profession—both urge the thorough removal of decay before filling the teeth—both recommend that the cavity be kept perfectly dry—both insist on a hard and compact plug—both talk learnedly about the effects of dead roots and aching teeth on the general health; yet I find by observation, that Mr. D. does not always get the diseased portion well removed—that it is apparently impossible for him to keep the saliva from entering faster than he can force the gold into the cavity; and, what is equally bad, I have noticed that his fillings look rather rough and spongy, and on examination with a sharp-pointed plugger, I find that a little force will carry the instrument through the plug. Dr. A.'s fillings are hard and polished; and I should judge from the general appearance of the teeth and plugs, that if both charged the same price per plug, that Mr. D. saves one-half his gold, and as much of his labor.

The same difference is apparent in the treatment of the mouth suffering from the old roots and aching teeth. Dr. A. manages to clear the mouth of these irritating causes of disease. Mr. D. perhaps removes one or two, and breaks off as many, when his patient, wearied out, gives up, and his mouth is worse than before.

I find, indeed, that it appears to be remarkably easy for almost any one to contend for much which is correct in dentistry, and that it is also a very easy matter for almost any one who has read Harris, or even Bell (and who have read nothing else appertaining to the profession,) to superficially palm himself off as a well-read Dentist. But I do not generally find all such practice, or even pretend to practice according to the principles laid down by either. I am not aware, however, that either of these gentlemen pretend to have written all relating to the science which is essential to be known; so far from this being the case, they evidently wish to impress the student with the fact, that the foundation principles of the science itself must be obtained from other books—that they only are building the superstructure; and hence they refer the students to works on Anatomy, Physiology, Pathology, Therapeutics, Chemistry, &c. &c. These all appear to be considered as important studies, at least so considered by most of our late dental authors; and not only these, but I have noticed that a majority of our practicing dentists begin to think so too. And I must confess, that I scarcely know a man in the profession, who has acquired any reputation at all, but takes this view of the matter; and if they have not previous to embarking in the profession made these branches of science their study, they have done so since.

If, therefore, these studies are essential, and observation appears to confirm this view, why is it that some of those advocating this course of study as important to the dental practitioner, do not require it of their students? Can these separate branches of science be mastered by any one in three or six months? Is not two years a short period for this herculean task? I believe our Dental Colleges have viewed this question in about this light. They, at least, require that candidates for graduation shall bear a satisfactory examination on all these collateral branches of dental science, as well as on operative and mechanical dentistry. They have either acted wisely, or they have not; and the question devolves itself on this proposition, viz: Are young men, thus educated, better prepared to discharge the duties of the profession, than those ignorant of all which these studies teach.

Let us take a very simple manner of testing this question. I will ask the mere mechanical operator what effect might possibly result from a diseased condition of the gums, when the teeth were beginning to loosen, and matter was formed around their necks.

I put this question because it is of common occurrence; and the only possible answer he can give *with his knowledge*, is, that the teeth will continue to loosen and drop out, unless the disease is arrested: observation may have led him to this conclusion.

It will be perceived that this answer only gives what is the most apparent consequence—the loss of the teeth to the patient. But, to the man of science, this may appear as the least important consequence as the result of the disease. He at once calculates the injury this local disease may have on the general health of his patient. He may indeed, at times, trace its origin to some other disease which is preying upon the system. He ascertains the chemical nature of the matter thus thrown off; the effect this may have when taken into the stomach;—and if he should find it the same—which may in some cases destroy the life of the dissector, which, in other cases, entering the circulatory system, vitiates the blood, induces hectic fever, impairs the digestive organs, and so deranges at times the nervous system as to cut short the life of his patient—he will only the more appreciate the importance of his profession, and feel the necessity of renewed diligence in acquiring all that knowledge which may contribute to aid him in alleviating "the ills to which flesh is heir."

By such comparisons as the above, might this subject be elucidated, until the whole field of Dental Practice is embraced, and in every particular we would find the ignorant pretender at fault. So apparent is this, that it has struck me as remarkable, that the intelligent part of the profession have not long since come to some general understanding on this subject, and marked out, as in medicine, some particular and definite course of study, which they at least should always exact of every student. It is to be hoped this soon will be the case. The intercourse brought about by the different associations for the promotion of dental science, now affords a fine opportunity for the accomplishment of this object.

Before I close these remarks, I wish particularly to draw the attention of the dental student to the importance of starting aright in his profession. The necessity that he commence his studies in the right manner, that his future professional career may not be embittered with the reflection that he assumed so important a duty without any proper qualification—that many of his operations, although mechanically well executed, must prove useless, or even injurious; thus bringing reproach upon himself and the profession, and, consequently, securing to himself the malediction of his unfortunate patrons.

It is here that quackery (the scourge of every profession) first makes its appearance; for intelligence, based upon correct principles, always holds aloof from her contamination. Ignorance, however, backed by "sordid *pelf*," takes her to its embrace, and, unfortunately, the alliance generally becomes so strong as to resist all after qualms of conscience which may urge the victim to shake off her foul embrace and come forth an independent freeman; consequently it is here the dental student should first learn to resist the enticements of profit and gain she holds out, and determine on acquiring a thorough knowledge of the profession, and that, too, before taking upon himself its duties. How seldom do we find those who have once engaged in dental practice, relinquish, even for a short time, the present prospect of even small gain, that they may prepare themselves by study to reap, ultimately, a rich and ample harvest. They will not or cannot appreciate the advantages of aught but what appears, to their narrow vision, as immediately applicable to the performance of that which has proved rather difficult to them in their art. That the general principles of medical science might benefit them, is beyond their conceptions; and, forsooth, because some of the reputable of the medical profession have winked at their ignorance, and flattered their vanity by praising their operations (of which by the by they are no judge,) they fain would think themselves as wise as the best, and far beyond improvement by any farther study.

> "A little learning is a dangerous thing;
> Drink deep, or taste not the Pyrean spring."

Their vanity may be so inflated that they cannot see the difference between ridicule and respect; and because the learned are too polite to treat them rudely, they regard not the necessity there is of thorough knowledge in their profession, that they may gain the esteem and hearty patronage of the intelligent.

It becomes *me* not to mark out a certain course of study to be pursued by all who may choose dentistry as their pursuit. The two institutions which profess to teach it as a science, have, perhaps, done this as well as is practicable at the present time. I have heard no one, in or out of the profession, who can lay any claim to knowledge on this subject, say that they require too much.

I apprehend that we are too far advanced in the nineteenth century to even suppose that a knowledge of Anatomy, Physiology, Pathology, Therapeutics, Chemistry, &c., &c., will injure any man who may engage in the practice of dentistry, or even that he is not by their aid better prepared to do justice to his patients. If this, then, be the case, it is the duty of every student to thus study, and thus prepare himself for the important responsibilities that will devolve upon him. None but he who has thus acquired the requisite qualifications, can form a correct diagnosis of the various diseases that occur about the buccal cavity, and the proper remedies for the judicious treatment of the same.

True knowledge cannot be communicated; it must be acquired, mastered, and made practical, or it is of but little avail. Thus obtained and appropriated by the Dentist, and he can with safety enter upon the duties of his profession, and feel that he, at least, will not disgrace the profession of his choice.

